# Overcoming insecticide resistance in *Anopheles* mosquitoes by using faster-acting solid forms of deltamethrin

**DOI:** 10.1186/s12936-023-04554-x

**Published:** 2023-04-21

**Authors:** Jessica Carson, Bryan Erriah, Stephania Herodotou, Alexander G. Shtukenberg, Leilani Smith, Svetlana Ryazanskaya, Michael D. Ward, Bart Kahr, Rosemary Susan Lees

**Affiliations:** 1 Vector Biology Department, Liverpool School of Tropical Medicine, Pembroke Place, Liverpool, L3 5QA UK; 2 Department of Chemistry and Molecular Design Institute, New York University, 29 Washington Place, New York, 10003 NY USA; 3 Liverpool School of Tropical Medicine, Innovative Vector Control Consortium, Pembroke Place, Liverpool, L3 5QA UK

**Keywords:** Deltamethrin, Pyrethroid, Resistance, Anopheles, Crystal structure, Crystal polymorphism

## Abstract

**Background:**

Controlling malaria-transmitting *Anopheles* mosquitoes with pyrethroid insecticides is becoming increasingly challenging because of widespread resistance amongst vector populations. The development of new insecticides and insecticidal formulations is time consuming and costly, however. A more active crystalline form of deltamethrin, prepared by heating the commercial crystalline form, previously was reported to be 12-times faster acting against susceptible North American *Anopheles quadrimaculatus* mosquitoes. Herein the potential for heat-activated deltamethrin dispersed on chalk to overcome various resistance mechanisms amongst five West African *Anopheles* strains is investigated, and its long-term sustained lethality evaluated.

**Methods:**

The more active deltamethrin form was generated in a commercial dust containing deltamethrin by heating the material as purchased. Tarsal contact bioassays were conducted to investigate its efficacy, potency, and speed of action against resistant *Anopheles* populations compared to the commercially available form of deltamethrin dust.

**Results:**

In all cases, D-Fense Dust heated to generate the more active form of deltamethrin was substantially more effective than the commercially available formulation. 100% of both Banfora M and Kisumu populations were knocked down 10 min post-exposure with no recovery afterwards. Gaoua-ara and Tiefora strains exhibited 100% knockdown within 15 min, and the VK7 2014 strain exhibited 100% knockdown within 20 min. In all cases, 100% mortality was observed 24 h post-exposure. Conversely, the commercial formulation (unheated) resulted in less than 4% mortality amongst VK7 2014, Banfora, and Gaoua-ara populations by 24 h, and Tiefora and Kisumu mosquitoes experienced 14 and 47% mortality by 24 h, respectively. The heat-activated dust maintained comparable efficacy 13 months after heating.

**Conclusions:**

The heat-activated form of commercial deltamethrin D-Fense Dust outperformed the material as purchased, dramatically increasing efficacy against all tested pyrethroid-resistant strains. This increase in lethality was retained for 13 months of storage under ambient conditions in the laboratory. Higher energy forms of commonly used insecticides may be employed to overcome various resistance mechanisms seen in African *Anopheles* mosquitoes through more rapid uptake of insecticide molecules from their respective solid surfaces. That is, resistant mosquitoes can be killed with an insecticide to which they are resistant without altering the molecular composition of the insecticide.

## Background

**Fig. 1:**
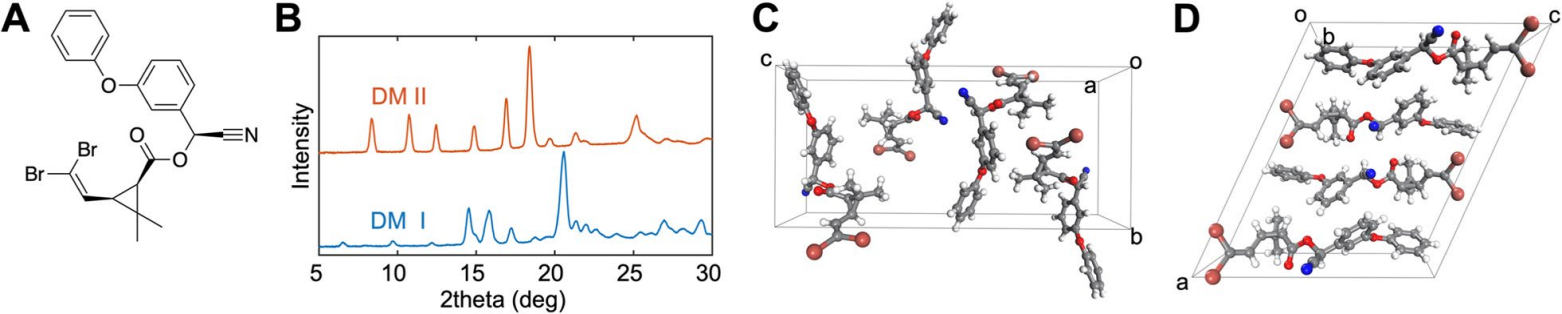
**A** Molecular structure of DM. **B** Powder X-ray diffraction data from Form I (blue) and Form II (orange). **C** and **D** Single crystal structures of Form (**C**) I and (**D**) II determined at 100 K. Figure adapted from Yang et al. [5]

Resistance to pyrethroids, until recently the only class of insecticides available for use on insecticide treated nets (ITNs), is widespread amongst *Anopheles* mosquitoes, the malaria vector. Resistance has greatly reduced pyrethroid efficacy and threatens the substantial progress made in malaria control in the twenty-first century [1, 2]. The extensive use of pyrethroids has resulted in different degrees of resistance and different resistance mechanisms amongst numerous populations despite the presence of geographical isolation. Deltamethrin (DM, (*S*)-cyano(3-phenoxyphenyl)methyl(1*R*,3*R*)-3-(2,2-dibromo-vinyl)-2,2-dimethylcyclopropane-1-carboxylate; Fig. 1), a synthetic pyrethroid insecticide the foremost active ingredient for indoor residual spraying (IRS), providing malaria control for millions [1, 3]. DM is an active ingredient in five IRS formulations, ten ITNs and two space sprays amongst World Health Organization (WHO)-listed prequalified vector control products [4]. DM is widely used for control of *Anopheles* vectors of malaria and *Aedes* vectors of arboviruses.

Contact insecticides, like pyrethroids, are often crystalline. Recent reports from the authors of this study have revealed that insecticidal activity of a contact insecticide depends on the crystal structures and associated free energies of its solid forms [5–9], also known as polymorphs. Different crystalline forms are often accessible under ambient conditions. The lethality of insecticides, including DM, relies on its uptake by the insect upon contact with crystal surfaces followed by migration of the molecular form of the insecticide to the target site. Resistance to an insecticide may be associated with different mechanisms, including mutations in the target site or upregulation of the cytochrome P450 enzymes involved in metabolism [10, 11]. It is reasonable to posit that an increased rate of insecticide uptake can overwhelm metabolic resistance mechanisms.

The relationship between the lethality of a crystalline contact insecticide and its crystal structure ultimately arises in thermochemistry. Weak intermolecular interactions and associated shallow potential energy hypersurfaces readily lead to solid crystalline forms (polymorphs) with different molecular organizations in the solid state, accompanied by distinct chemical and physical properties including crystal free energies. An inverse correlation between lethality and thermodynamic stability of polymorphs was first reported in historically relevant compounds such as DDT and lindane, but also for contemporary insecticides in the form of newly discovered polymorphs of DM and imidacloprid, which are amongst the most widely used insecticides today. Seven new polymorphs of imidacloprid were discovered in addition to two previously reported forms [6]. These polymorphs differ with respect to the arrangement of the molecules in their crystal lattices and their presentations at distinct crystal surfaces. Notably, the least stable polymorph of imidacloprid was six and nine times more active against susceptible *Anopheles* and *Aedes* mosquitoes, respectively, than the commercial, thermodynamically stable, form. Importantly, these metastable polymorphs were found to be stable against transformation to the thermodynamically stable form for at least 6 months, meeting WHO guidelines for practical use in the field [6, 12]. A second DM polymorph (Fig. 1) [5], denoted Form II after determination of its crystal structure, was generated by cooling of its melt. Crystals of Form II were nearly seven times more active than Form I against *Drosophila melanogaster*, an accepted proxy for vectors [13]. Notably, a commercial dust formulation containing DM (D-Fense Dust; 0.05 wt% DM) that was heated above the DM melting point and then allowed to cool to ambient temperature was 9 and 12 times faster-acting than the original DM dust against susceptible *Aedes aegypt*i and *Anopheles quadrimaculatus* mosquitoes, respectively. This increased lethality was similar to that observed for pure Form II against *Drosophila*, consistent with the more active form in the dust. Moreover, this heightened lethality, which was sustained for at least 3 months, indicated that a more active metastable form could be kinetically stable against transformation to a less active thermodynamically stable form [5]. Whilst the molecular structure of deltamethrin (Fig. 1A) remains unaffected by this simple operation, the specific arrangement of the molecules in the crystal lattice is different for the two forms, as deduced from powder X-ray (Fig. 1B) and single-crystal diffraction (Fig. 1C, D). New insecticides, repellents and anti-malarial compounds have been introduced in recent years, but the introduction of new chemical agents in the field requires sizeable investments of labour and capital [14–17]. Consequently, the engineering of more active solid forms of existing chemical compounds through manipulation of their crystal structure can be faster, less expensive, and less risky because new compositions of matter are obviated [18].

Herein, the tarsal assay method is used to demonstrate that the heat-activated DM dust is highly effective against resistant *Anopheles coluzzii* strains Banfora M, VK7 2014, and Tiefora, as well as the *Anopheles arabiensis* strain Gaoua-ara [19]. *Anopheles gambiae* is a complex of mosquito species, three of which are associated with high pyrethroid resistance in Burkina Faso including *An. gambiae sensu stricto* (*s.s.*), *Anopheles coluzzii*, and *An. arabiensis* [18]. These results reveal that high energy solid forms of contact insecticides can overwhelm certain resistance mechanisms because of rapid insecticide uptake, suggesting a pathway to tackling vector control among highly resistant populations of mosquito.

## Methods

### Tarsal assays

D-Fense Dust, purchased from Pestcontrolpros, consists primarily of a calcium carbonate carrier on which deltamethrin (DM) was dispersed at a loading of 0.05 wt%. Dust was activated by heating to 150 ℃ for 30 min in a glass vial, and then allowed to cool to ambient temperature. Tarsal plate assays were prepared by dispersing 0.05 g of this heat-activated D-Fense Dust onto 100 mm tarsal plates alongside an equal number of plates to which the unheated D-Fense Dust was dispersed in the same manner. Ten mosquitoes then were introduced to each plate. Mosquitoes were exposed for 30 min, during which knockdown (KD), averaged over three trials, was scored at five-minute intervals. Knockdown was also scored at 60 min and mortality at 24 h post-exposure. Control plates containing no D-Fense Dust also were tested for comparison. The amount of dust on each tarsal plate corresponds to an estimated DM dosage of approximately 80 mg/m^2^, based on 0.05% loading of DM in the dust. Tarsal plate assays to determine the activity of the D-Fense carrier dust alone were performed with commercial dust that was heated at 500–550 °C for 30 min, a treatment which caused deltamethrin to be decomposed, as determined from thermogravimetric analysis of pure deltamethrin. The D-Fense dust heated at 500–550 °C showed no DM traces above noise when tested by mass spectrometry (Triple Quadrupole Mass Spectrometry); the organics were pyrolysed.

### Mosquito rearing

The Kisumu strain of *An. gambiae* was colonized in Kenya in 1975. The strain held at the Liverpool School of Tropical Medicine (LSTM) was sourced from the Malaria Research and Reference Reagent Resource Centre (https://www.beiresources.org/About/MR4.aspx). The VK7 2014 strain from the Houet Province, Burkina Faso Valley de Kou 7 and the Banfora M strain from the Comoé Province, Banfora district, Burkina Faso, were colonized from larval collections by the Liverpool School of Tropical Medicine (LSTM) and the Centre National de Recherche et de Formation sur le Paludisme (CNRFP) in 2015. The Tiefora strain from Comoé Province, Banfora district, Burkina Faso was colonized from larval collections by LSTM and CNRFP in 2018. The Gaoua-ara strain from Poni Province, Gaoua district, Burkina Faso was colonized from larval collections by the Institut de Recherche en Sciences de la Santé (IRSS) in 2018.

All resistant strains (all but Kisumu) were routinely selected every 3^rd^ to 5^th^ generation by exposure to 0.05% deltamethrin in a WHO cylinder bioassay to maintain their resistant phenotype, and all strains were profiled annually against eight insecticides (except VK7 2014 which was profiled against six insecticides) from different mode of action classes [19]. Gaoua-ara, Banfora M, Tiefora and VK7 2014 were shown to be resistant to pyrethroids and DDT, Gaoua-ara and Tiefora were also resistant to dieldrin, propoxur and bendiocarb, and Banfora M to bendiocarb. Significant PBO synergism in all resistant strains indicates the involvement of metabolic resistance. Target site mutations are present in the resistant strains in different combinations and frequencies, and there is upregulation of other genes implicated in insecticide resistance [19]. All mosquitoes were reared under standard conditions at 26 ± 2 °C and 70% relative humidity ± 10% under L12:D12 h light: dark photoperiod. Larvae were fed on ground fish food (TetraMin^®^ tropical flakes, Tetra®, Blacksburg, VA, USA) and adults were provided with 10% sucrose solution *ad libitum*.

## Results

**Fig. 2:**
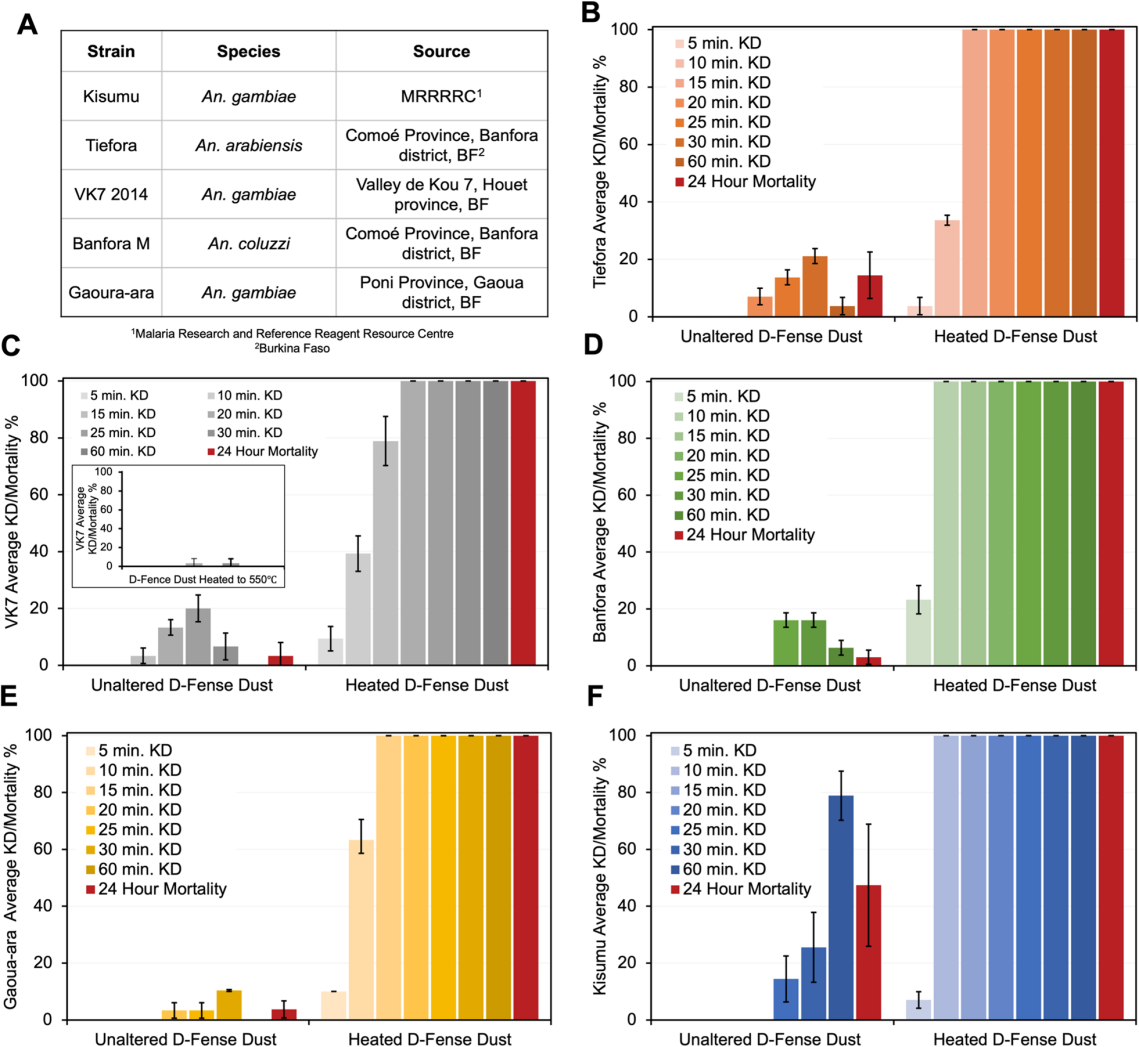
**A** species, strain and origin of mosquitoes used in this study. **B–F** WHO tarsal assay. Average knockdown % and 24 h hour mortality of pyrethroid-resistant *Anopheles* strains **B** Tiefora, **C** VK72014, Inset C. WHO tarsal assay. Average knockdown % and 24 h hour mortality of pyrethroid-resistant *Anopheles* VK7 2014 strain exposed to commercial D-Fense Dust heated to 500–550 °C. **D** Banfora M, **E** Gaoua-ara **F** Kisumu *Anopheles* mosquitoes exposed to heated D-Fense dust (150 °C, 30 min). The errors bars correspond to one standard deviation based on triplicate measurements

The inefficacy of commercial deltamethrin D-Fense Dust against resistant strains was apparent by comparison to the heat-activated dust (Fig. 2B–F). Tarsal assay data were collected by exposing five resistant strains of *Anopheles* mosquitoes to both the commercial D-Fense Dust as obtained and the heat-activated formulation. Control plates without D-Fense Dust revealed a negligible number of mosquito deaths, within the statistical error of the companion measurements. In DM dusts heated to 500–550 °C, the DM crystallites admixed in the chalky carrier were fully decomposed after heating as shown by TQD (Triple Quadrupole) mass spectrographs that were absent of the DM parent ion (505.9 m/z). The heated dusts were not lethal (Fig. 2, Inset C), confirming that deltamethrin was the primary contributor to insect death.

Commercially obtained D-Fense Dust was ineffective against Tiefora mosquitoes (Fig. 2B) until 20 min, the first observation of knockdown, at which ca. 7% were knocked down. A peak in knockdown (21%) was observed at 30 min, but after 24 h total mortality was lower (14%). Similar trends were observed for the other strains tested. The initial knockdown for VK7 2014 (Fig. 2C) was 3% at 15 min, peaking at 20% at 25 min, but with only 3% mortality after 24 h. Initial knockdown amongst Banfora mosquitoes (Fig. 2D) was observed at 25 min, where a peak 16% knockdown was recorded. Only 3% mortality was observed amongst Banfora mosquitoes after 24 h, however. The first observation of knockdown amongst Gaoua-ara mosquito (Fig. 2E) populations was 3% knockdown at 20 min with a peak of 10% knockdown at 30 min, but only 3% mortality after 24 h. Kisumu mosquitoes (Fig. 2F) were more affected by the commercial product than the other four strains. The first observation of knockdown was at 25 min (14%), with peak knockdown (79%) seen at 60 min. After 24 h mortality was recorded as 47%.

The heat-activated D-Fense Dust resulted in 100% mortality 24 h post-exposure for all five strains tested. Knockdown was first observed after 5 min of exposure amongst Tiefora (Fig. 2B) populations, with 100% knockdown at 15 min and 100% mortality at 24 h. Similarly, VK7 2014 (Fig. 2C) showed 9% knockdown by 5 min, 100% by 20 min, and 100% mortality at 24 h. Notably, 100% knockdown was observed later amongst VK7 populations than in other populations tested. Banfora and Kisumu (Fig. 2D and F) strains both exhibited 23% and 7% knockdown at 5 min, respectively. Both strains had 100% of the population knocked down by 10 min, and 100% mortality at 24 h. 10% of Gaoua-ara mosquitoes (Fig. 2E) were knocked down by 5 min, and 100% by 15 min, and much like the other strains, had 100% mortality 24 h.

**Fig. 3:**
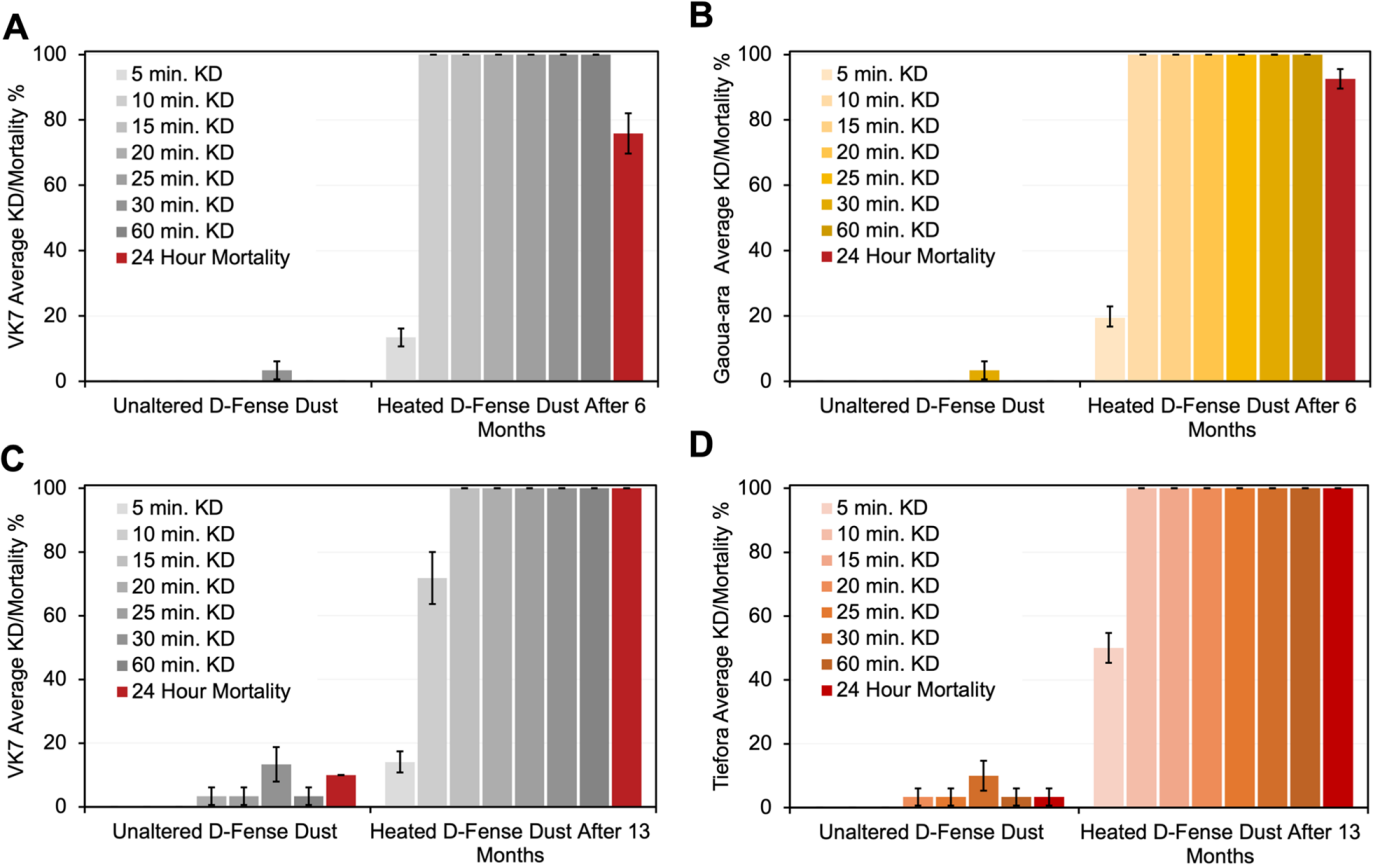
WHO tarsal assay. Average knockdown % and 24 h hour mortality of *Anopheles* mosquitoes exposed to D-Fense Dust that was heated 6 months (**A** and **B**), and 13 months prior (**C** and **D**). **A**, **C** VK7 2014, **B** Gaoua-ara, **D** Tiefora

The lethality of the heat-activated dust was tested after storage for 6 months against VK7 2014 and Gaoua-ara *An. coluzzi* and *An. arabiensis*, respectively, and then again at 13 months against VK7 2014 and Tiefora *An. coluzzi* to evaluate the sustained lethality after storage (Fig. 3A–D). Against VK7 2014 and Gaoua-ara, the D-Fense Dust, used as obtained, was virtually ineffective, in both cases exhibiting knockdown only at 30 min (3%), and 0% mortality after 24 h. Conversely, the heat-activated D-Fense Dust maintained its lethality after 6 months. Against both strains, the first observation of knockdown was observed after 5 min, 19% in Gaoua-ara and 13% in VK7 2014. After 24 h, mortality amongst the VK7 2014 and Gaoua-ara are observed to be 76% and 92%, respectively. Notably, again, the VK7 2014 strain remained slightly less affected by the heat-activated dust than other strains, with over 20% of knocked down mosquitoes recovering by 24 h post-exposure. After 13 months, the heat-activated D-Fense Dust maintained its lethality (Fig. 3C and D), and the first observation of knockdown was observed after 5 min, 14% in VK7 populations and 50% in Tiefora populations. After 24 h, mortality amongst the VK7 2014 and Tiefora is observed to be 100% in both cases. These data demonstrate that the more lethal solid form of deltamethrin produced by heating is stable against transformation to the thermodynamically stable, and less active, form for at least 13 months, meeting WHO guidelines for practical use in the field. This observation should encourage the use of metastable forms of contact insecticides for control of malaria vectors [14].

## Discussion

In lieu of directly monitoring the path of molecules from crystals to target site, the assays detailed in Fig. 3B–F and A–D provide an insight into the kinetics associated with the differential rates of uptake of solid forms by mosquito populations, and how this determines the overall efficacy of a contact insecticide. Against all strains, heat-activated D-Fense Dust was faster acting than the commercial form and eventually also substantially more lethal, as observed by 24-hour mortality. This demonstrates that the rate of uptake, while being often overlooked, is an important part of a larger dynamic.

The difference in efficacy between the heat-activated and commercial dusts against VK7 2014 mosquitoes is less pronounced at early exposure times than observed for other strains. After 24 h, however, a substantial difference is evident; less than 5% of the population is killed by the unheated dust whilst 100% of the population is killed by the heat-activated dust. The difference between VK7 2014 and the other strains is highlighted by the longer time taken to reach 100% knockdown. Whereas VK7 2014 reaches 100% knockdown in 20 min, all other strains reach 100% knockdown within 10–15 min. When exposed to dust heated 6 months prior, some recovery of knocked down mosquitoes was observed at 24 h in Gaoua-ara (8%), but a greater proportion in VK7 2014 (24%). This again indicates a slightly lower efficacy against this strain. Naturally, different resistance mechanisms detoxify and/or nullify insecticides differently, and this is revealed by experiments featuring resistant VK7 2014 mosquitoes (Figs. 2C and 3A). The combination of resistance mechanisms expressed by each resistant strain is different and it is difficult to determine the relative contribution of each or explain this difference. VK7 2014 is fixed for the 995 F allele, whereas this SNP is present at low frequencies in the other strains tested which may play a role, as may an elevated level of cuticular hydrocarbons [19, 20]. After 13 months, no recovery of knocked down mosquitoes was observed at 24 h, likely owing to slight differences in degrees of resistance between populations.

In addition to target site and metabolic resistance mechanisms, there are mechanisms present in the intensely pyrethroid resistant strains tested here that may directly affect the uptake and penetration of insecticide. Epicuticular thickening has been observed to reduce penetration by insecticides and contribute to pyrethroid resistance [21–23]. The chemosensory protein SAP2 is expressed in mosquito legs and antennae and overexpression has been linked to pyrethroid resistance in another resistant strain of *An. gambiae sensu lato* (*s.l*.) from Burkina Faso [24]. Genes implicated in the cuticular hydrocarbon (CHC) synthetic pathway are upregulated in Banfora M, Bakaridjan and Gaoua-ara strains, though downregulated in Tiefora and VK7 2014, and it seems that cuticular resistance may be particularly significant in Gaoua-ara [19].

Using a commercially available dust product, sold for the control of household pest insects, this study provides a dramatic demonstration of the potential to overcome pyrethroid resistance mechanisms by manipulating the properties of crystalline insecticides. There is a very limited number of insecticides available for use in public health, and the discovery and development of new compounds is slow and expensive. With so few tools in the toolbox, it is critical that none are discarded prematurely. These results suggest that it may be possible to produce more efficacious forms of pyrethroid insecticides to overcome existing resistance mechanisms, and so restore and retain the usefulness of this historically important class of insecticides. As new insecticides are developed it is critical that each is used judiciously to slow the rate at which resistance evolves. Further investigation is warranted for a better understanding of how crystalline insecticides are presented to mosquito vectors of disease under other relevant conditions, for example on the fibers of an ITN or in an IRS formulation. By discovering the effect of changes in the physical presentation and establishing the most efficacious form of each insecticide on a given substrate, both at the product development stage and throughout the product lifecycle, it may be possible to reduce the amount of insecticide which is needed for a product to be effective. This will be particularly important for novel compounds which may be considerably more expensive than pyrethroids. It is important to rethink the existing logic that how well a product kills a mosquito is determined simply by the concentration of insecticide present, and instead to consider its physical properties and presentation to potentially achieve a greater killing effect at a lower concentration.

## Conclusion

The results herein clearly show that a metastable form of deltamethrin is effective at overcoming some resistance mechanism or mechanisms found in pyrethroid-resistant mosquito populations. Against all resistant strains that were tested, the heat-activated dust was vastly superior. Heat-activated D-Fense Dust killed 100% of each strain by 24 h post-exposure, whereas the highest mortality observed from the commercial form was approximately 14%. Heat-activated dust acted slowest against VK7 2014 mosquitoes, but mortality was still high showing that enhanced effects are seen across a range of resistant strains, and in turn mechanisms and combinations of mechanisms. The heat-activated dust maintained comparable efficacy 13 months after heating. Previously, discovery of a faster acting form of deltamethrin was reported which showed its higher lethality against *Anopheles* and *Aedes* mosquitoes when compared to the commercial form [5] Now, the same faster acting form has also been shown to be faster acting and more lethal against pyrethroid-resistant strains, thus highlighting the pivotal role that solid-state chemistry must play in the fight against insecticide resistance in mosquito populations. There are very few insecticides of different mode of action classes available for use against mosquito vectors of disease; until recently only pyrethroids were used in ITNs. Though ITNs containing a pyrethroid plus chlorfenapyr, pyriproxyfen or the synergist piperonyl butoxide (PBO) are now available [4], the development pipeline for new and even repurposed insecticides is long and vastly expensive. The formulation of DM or other pyrethroids in a form able to overcome resistance may provide an effective new tool in a much shorter period. Most of the known and novel insecticides are crystalline and understanding how their crystalline structure could affect bioactivity is critical for the development of the best possible vector control products. Greater knowledge of the crystallization behaviour of insecticides and the application of knowledge from crystal engineering could help to overcome resistance [25]. It could also reduce the cost of vector control products, allowing smaller quantities of compound to be used in its most efficacious physical state to greater effect.

## Data Availability

The datasets generated and/or analysed during the current study are available from the corresponding author on reasonable request.

## Abbreviations

CHC: Cuticular hydrocarbon
DM: Deltamethrin
IRS: Indoor residual spraying
ITNs: Insecticide treated nets
KD: Knockdown
LLINs: Long lasting insecticidal nets
PBO: Piperonyl butoxide
